# Altered Autonomic Function in Metabolic Syndrome: Interactive Effects of Multiple Components

**DOI:** 10.3390/jcm13030895

**Published:** 2024-02-03

**Authors:** Joseph Mannozzi, Louis Massoud, Jon Stavres, Mohamed-Hussein Al-Hassan, Donal S. O’Leary

**Affiliations:** 1 Department of Physiology, Wayne State University School of Medicine, Detroit, MI 48001, USA; 2 School of Kinesiology and Nutrition, University of Southern Mississippi, Hattiesburg, MS 39406, USA

**Keywords:** MetS, exercise, autonomic function, obesity, hypertension, baroreflex, metaboreflex

## Abstract

Metabolic syndrome (MetS) describes a set of disorders that collectively influence cardiovascular health, and includes hypertension, obesity, insulin resistance, diabetes, and dyslipidemia. All these components (hypertension, obesity, dyslipidemia, and prediabetes/diabetes) have been shown to modify autonomic function. The major autonomic dysfunction that has been documented with each of these components is in the control of sympathetic outflow to the heart and periphery at rest and during exercise through modulation of the arterial baroreflex and the muscle metaboreflex. Many studies have described MetS components in singularity or in combination with the other major components of metabolic syndrome. However, many studies lack the capability to study all the factors of metabolic syndrome in one model or have not focused on studying the effects of how each component as it arises influences overall autonomic function. The goal of this review is to describe the current understanding of major aspects of metabolic syndrome that most likely contribute to the consequent/associated autonomic alterations during exercise and discuss their effects, as well as bring light to alternative mechanisms of study.

## 1. Introduction

### 1.1. Metabolic Syndrome: Clinical Presentation and Effects

There are many well-studied metabolic disarrangements that are known to negatively impact cardiovascular health such as obesity, hyperlipidemia, insulin resistance, diabetes, and hypertension. When present collectively, these cardiovascular and metabolic alterations comprise an entity termed metabolic syndrome (MetS). The diagnostic criteria for MetS defined by the National Heart, Lung, and Blood Institute are as follows: obesity, low HDL, elevated blood pressure, elevated fasting glucose, and high triglycerides (Table 1).

The diagnosis of MetS is made when three out of five diagnostic criteria are present. The likelihood of being diagnosed with MetS is increasing at an alarming rate, especially considering that 573 million individuals are projected to be obese by 2030 [1,2], and about half of the U.S. population already suffers from some form of hypertension [3]. Both obesity and hypertension modify autonomic function, manifesting as exaggerated muscle metaboreflex responses and altered baroreflex function [4,5,6,7,8,9,10,11,12,13,14,15,16,17,18,19,20,21,22,23]. While obesity and hypertension are only two of the risk factors of MetS, diabetes, dyslipidemia, and insulin resistance all play a role, either through modification of sympathetic function [6,24,25,26,27,28] or through the development of atherosclerotic disease and alterations in metabolic homeostasis. Indeed, obesity and hypertension likely provide common soil for the development of diabetes and coronary vascular disease [29]. All these risk factors could enhance metabolite-sensitive reflexes, such as the muscle metaboreflex, or induce attenuation of baroreflex responsivity and sympathetic control. Exaggeration of muscle metaboreflex responses and/or attenuation of the arterial baroreflex, which buffers muscle metaboreflex responses, likely leads to exercise intolerance and potentially cardiovascular complications during exercise. Thus, the focus of this article will be to discuss the current and potential effects of MetS components on the neural control of cardiovascular function during exercise, with a particular focus on arterial baroreflex and muscle metaboreflex function.

### 1.2. Current Challenges to Animal Models of Metabolic Syndrome

Each of the major components of MetS, namely obesity, pre-diabetes, and hypertension, have been shown to independently influence sympathetic control. Hypertension in isolation significantly alters sympathetically-mediated arterial baroreflex [14,30,31,32] and muscle metaboreflex responses [14,15,17,18,19,32] during exercise. The impact that obesity alone plays is less understood, having been shown to either inhibit or enhance sympathetic activity [4,5,23,33,34]. Diabetes, when uncontrolled, has been shown to induce varying levels of neuropathy that, in turn, induce alterations in autonomic function. However, others have found that insulin alone can be a potent initiator of sympathetic responses prior to the development of type 2 diabetes [24,35,36]. Long-term dyslipidemia and triglyceride storage have been linked to the development of peripheral artery disease, which can initiate changes in autonomic function through physical alterations in the peripheral vasculature such as the development of atherosclerotic lesions and arterial stiffening which, in turn, can enhance sympathetically mediated responses to high metabolic load. Thus, each MetS component can independently inflict alterations in autonomic function, which ultimately provides a difficult challenge to maintain exercise tolerance and performance.

Adding an additional degree of complication to the study of autonomic function in MetS is that the observed phenotype of MetS patients is not uniform in their development or presentation. For instance, some individuals may be diagnosed with MetS using the minimum three criteria, while others may have four or all five components of MetS. Furthermore, within these varying diagnoses, the criteria in which a diagnosis of MetS is met can vary as well, some patients may exhibit hypertension, elevated triglycerides, and elevated fasting glucose, whereas another patient diagnosed with MetS can exhibit obesity, dyslipidemia, and elevated fasting blood glucose. Thus, the interplay between each of the components that compromise the phenotype of each MetS patient are important considerations in the understanding of alterations in autonomic function. Thus, with each MetS component having the potential to contribute to altered autonomic states several questions arise: First, which components, if any, are the most potent effectors in altering autonomic function? Second, does the accumulation of multiple components exert additive, or redundant influences on autonomic dysfunction? And finally, how might the removal of select MetS components influence the observed autonomic phenotype?

These questions are difficult to answer. MetS is challenging to study in animal models due to the complexity of the disease processes. Although animal models of MetS can closely mimic the risk factors and symptoms observed in humans, often, they do not consider the multifactorial entities that can lead to the development of the syndrome. In other words, beyond human studies, it may be inaccurate to say that animal models study “metabolic syndrome”, but rather that they fragment the disorder into its parts: hypertension, hypertension and insulin resistance, obesity and prediabetes-diabetes, or any other combination or singular aspect of the syndrome. The isolation of these MetS components is most common in monogenic or pathologic-bred rodent models, wherein aspects of the disease can be induced and observed in a more controlled manner without confounding the effects of other factors. While the monogenic models can control for various factors such as genetic predispositions and differences in disease severity, these models do not translate well into human models, as MetS is a multifactorial condition that varies in severity, genealogical interaction, as well as symptom presentation (i.e., the order in which symptoms originate or are added). The diet-induced obesity (DIO) models more closely mimic human models, as a surplus of caloric intake and an unhealthy diet drives the development of obesity and other symptoms such as diabetes and dyslipidemia and in some cases even hypertension [37]. However, this approach does not always lead to a true MetS phenotype in every animal model, and most studies that utilize diet approaches do not begin their study until the entirety of desired components have arisen. The phenotypic differences in the production of MetS models are driven by variability within the choice of diet to achieve the same end goal of utilizing caloric excess or glucose metabolism manipulation to elicit the components of MetS. de Moura e Dias et al. [38] discussed this variability in a literature review regarding the range of diets used to achieve MetS. For example, in some of these diets, lipids from calories ranged between 41–60%, and the choice of lipids in high-fat diets was saturated fatty acid in some studies but not in others [38]. It has also been reported that MetS can be induced with a hypercholestrolemic diet (with various percentages of highly saturated fat, cholesterol, and other fat), sucrose, and fructose [39]. It is important to note that these diets may not induce all the criteria that comprise MetS. For example, they may induce obesity or diabetes but not hypertension—a condition known to significantly alter autonomic function at rest and during exercise [37]. Only a select number of investigations have been able to successfully induce a comprehensive animal model of MetS, primarily by implementing variations or combinations of established diets [37]. Furthermore, in most studies, the goal is to study the final phenotype of MetS and not evaluate the various components of the syndrome as they arise to evaluate their overall contributions to the disease. In addition to these limitations within the current studies, the evaluation of autonomic function has been limited even in models that utilized MetS phenotypes that encompassed all components.

This variability in diet to achieve the end goal of inducing obesity, while useful in further understanding the complex pathophysiology of MetS, does not realistically mirror what occurs in humans nor does it permit the understanding of how the autonomic alterations induced by these components interact with each other. This is because the disease is not a frank appearance of symptoms all at once, but rather, an accumulation of metabolic disarrangement that leads to the development of factors over time. Therefore, this review makes a call for future research to move beyond evaluating the frank syndrome and more thoroughly examine the development of MetS characteristics during the diet-feeding process to determine each symptom’s contribution to the overall dysfunction of the syndrome.

## 2. Metabolic Syndrome and Hypertension

Hypertension is an important risk factor for MetS, with some studies suggesting a higher prevalence of MetS in hypertensive males and lower in females [40]. Although hypertension is a major factor regarding MetS, not all initial diagnoses of MetS contain hypertension as a component, and, in some cases, it is developed later as syndrome severity increases [40,41,42,43,44,45,46]. There are many suggested mechanisms that explain how components of MetS can induce hypertension, sometimes prior to an official diagnosis of MetS. Aggregation of adipose tissue with weight gain and obesity leads to the release of adipocytokines, including leptin, tumor necrosis factor (TNF)-α, resistin adiponectin, angiotensinogen, and fatty acids [47]. These affect vascular function through proinflammatory actions and induce oxidative stress, eventually contributing to the development of hypertension. Elevations in sympathetic activity combined with a hyperadrenergic state of inflammation and obesity have been shown to contribute to increases in blood pressure [48], and thus, may be the pathway by which initial symptoms of MetS lead to the development of additional components such as hypertension.

Conversely, some data suggests an almost identical whole-body sympathetic activity between obese and lean normotensive patients, suggesting that the initial symptoms of MetS may not be sufficient to induce neurogenic hypertension [10,21,23,49]. However, when these same models are assessed for regional alterations in norepinephrine spillover, differences are observed [10]. Regional differences have been shown to be important, as certain types, such as rearrangement of renal NE spillover in the context of obesity, may be a driving factor of hypertension in the early stages of the development of MetS [10].

Sympathetic overactivation is present within the context of hypertension in MetS [8,11,50]. Whether hypertension is the initial cause or an amplifying symptom of this phenomenon—or both—is yet to be determined. However, evidence does show that patients with MetS have increased sympathetic activity even without hypertension, and compared to hypertension alone [11]. Interestingly, as reviewed by Esler et al. [51], muscle-sympathetic nerve activity in patients with hypertension also seems to be further exaggerated as more components of MetS are present, indicating a compounding influence on disease progression.

Considering both hypertension and MetS manifest negative effects on cardiovascular health, it is important to recognize any prognostic value that may arise from understanding their relationship to one another. Schillaci et al. [43] showed that in association with high blood pressure, hypertensive subjects with MetS had an augmented cardiovascular risk, i.e., MetS was an independent risk factor for adverse cardiovascular events. This has also been shown in hypertensive patients at low-to-medium risk, where patients with hypertension and MetS were at a higher cardiovascular risk than patients with hypertension alone [52]. Being that MetS-related cardiovascular risk increases with the diagnosis of hypertension, the implications for strenuous bouts of activity are also of concern, as hypertension alone is known to significantly alter cardiovascular responses to exercise and increase the risk of cardiovascular ischemic events.

## 3. Autonomic Function: Hypertension

Both hypertension and MetS have been linked to autonomic dysfunction and exercise intolerance, [14,15,16,17,18,19,22,23,32,53]. Hypertension has been studied extensively in the context of exercise; hypertension modifies the muscle metaboreflex and the arterial baroreflex as well as their interactions [15,17,18,19,32,54,55]. Studies in humans have shown that activation of the muscle metaboreflex via post-exercise circulatory occlusion (which entraps metabolites in muscle during the recovery from exercise) evokes a larger increase in peripheral sympathetic activity [56,57] in hypertensive individuals. Similar conclusions were drawn using a decorticate rat model and electrically induced static muscle contraction or arterial infusion of substances that activate skeletal muscle metabolite-sensitive afferents [17,58,59,60,61]. In conscious dogs as well as human subjects, muscle metaboreflex activation during submaximal dynamic exercise increases arterial pressure predominately via reflex increases in cardiac output via substantial increases in heart rate, left ventricular inotropic state, and central blood volume mobilization despite large increases in ventricular afterload and arterial elastance [62,63,64,65,66,67,68]. After induction of hypertension, the ability to raise cardiac output is markedly reduced and the reflex shifts toward increased peripheral vasoconstriction [18]. Even the coronary circulation is vasoconstricted due to the heightened increase in sympathetic activity [18]. After the alpha adrenergic blockade, this metaboreflex-induced coronary vasoconstriction in hypertension is reversed to vasodilation and the ability to raise the inotropic state and cardiac output is returned to normal [18]. In contrast, the strength of the arterial baroreflex is reduced in hypertension [12,30,32,54,69,70,71]. The arterial baroreflex buffers the muscle metaboreflex thereby limiting the rise in arterial pressure [72]. This buffering occurs via arterial baroreflex inhibition of metaboreflex-induced peripheral vasoconstriction [73]. After arterial baroreceptor denervation, muscle metaboreflex activation induces substantial increases in both cardiac output and peripheral vasoconstriction which elicits marked increases in arterial blood pressure [73,74]. With the reduction in baroreflex function in hypertension, the larger sympatho-activation during metaboreflex activation could stem from reduced baroreflex buffering of the metaboreflex-induced increases in peripheral sympathetic activity. Thus, any interventions that improve baroreflex function (e.g., exercise training) [31,69,75,76,77,78,79,80,81], could serve to limit the rise in sympathetic activity during exercise and thereby lessen cardiovascular risk factors in hypertensive individuals.

Similar effects during exercise are observed regarding the impact of MetS on muscle metaboreflex activation, in which an enhanced vasoconstrictor response is observed similar to what has been seen in hypertension [22,23,53,82]. Furthermore, studies assessing the impact of obesity have shown that obesity alone does not significantly alter hemodynamics [23]. Thus, symptoms such as hypertension likely play a significant role in the autonomic alterations induced by MetS.

## 4. Metabolic Syndrome and Impaired Glucose Control

Elevated blood sugar is one of the diagnostic criteria of MetS. This is especially relevant in the context of the modern diet and eating habits, particularly as the 2017–2018 NHANES Study by the USDA showed that U.S. adults consume an estimated 17 tablespoons of added sugars per day. Insulin resistance has been implicated in playing a role in the development of MetS [83]. This means that insulin resistance increases cardiovascular risk and promotes the progression of cardiovascular disease [44]. Glucose intolerance is also correlated with impaired lipolysis [84] which may lead to alterations in cholesterol and free fatty acid concentrations observed in MetS. The Centers for Disease Control estimates that 90% of patients with diabetes meet the criteria for overweight or obesity and the relationship between insulin resistance, obesity, and MetS is an important one, especially with abdominal obesity also being one of the diagnostic criteria of MetS.

Type 2 diabetes is one of the more concerning factors of MetS similar to that of hypertension as once the process of glucose and insulin dysregulation begins the ability to appropriately correct toward baseline levels of glycemic control diminishes rapidly [85,86]. The damage initiated to pancreatic beta cells through the development of type 2 diabetes is irreversible [27,85,87,88,89,90], and unfortunately lays the groundwork for the development of other factors of MetS likely through the effects of insulin [6,26,36,91,92]. The only current therapies available for type 2 diabetes are mechanisms to control the levels of insulin production and effectiveness or reduction in glycemic load through pharmaceutical actions, exercise, and diet. Although glycemic control seems the best mechanism for enhancing the function of residual insulin production and action, for many, diet and exercise do not suffice or are not well tolerated either due to poor adherence to glycemic control plans, or exercise intolerance. Thus, pharmaceutical intervention is typically combined with diet and exercise as a primary treatment mechanism. To what degree type 2 diabetes or even prediabetes contributes to the development of MetS, and subsequent sympathetic dysregulation is unknown, as both conditions have the propensity to be precursors and developed components of MetS as the disease progresses. What is known is that insulin regulation seems to be a key factor.

## 5. Autonomic Function: Type 2 Diabetes

The effects of insulin resistance do not end with diabetes and obesity as discussed above since essential hypertension is linked to insulin resistance [92,93]. Considering that hypertension is partially a result of sympathetic overactivation, it is unsurprising that insulin plays a role in modulating sympathetic centers in the brain. Low plasma levels of insulin in a fasting state activate inhibitory pathways that suppress sympathetic centers in the brain stem which have been active chronically. This results from reductions in glucose metabolism in insulin-dependent hypothalamic neurons [94]. Thus, in conditions such as insulin resistance or early type 2 diabetes, increased insulin levels likely contribute to enhanced sympathetic activity at rest and during exercise and may play a potentiating role in the development of hypertension in MetS.

Type 2 diabetes has been shown to significantly modulate muscle metaboreflex-induced peripheral vascular responses by way of enhancing muscle sympathetic nerve activity and the peripheral vasoconstrictor response similar to what is observed in hypertension [16,95,96,97]. Furthermore, insulin itself, either in the context of diabetes development or via hyperinsulinemia, also exerts an effect on autonomic reflexes, such as the arterial baroreflex [98,99]. Pricher et al. [100] reported that lateral ventricular insulin infusion in rats increased baroreflex control of both heart rate and lumbar sympathetic nerve activity. Insulin infusion altered the baroreflex within 60–90 min, and a fourth ventricle infusion had no effect on the baroreflex. Thus, they concluded that insulin modulates baroreflex control of lumbar sympathetic nerve activity and heart rate at the level of the forebrain [100]. Ryan et al. [101] showed that in humans, cerebral blood flow possibly mediates the relationship between baroreflex sensitivity and insulin resistance. The regions in the central autonomic network include forebrain regions such as the pregenual anterior cingulate cortex and insula [101,102,103]. Other studies provided insulin resistance as a possible mechanism for impaired baroreflex gain [25,104]. Such findings make it clear that patients with MetS who develop insulin resistance and diabetes can also develop autonomic dysregulation. This in turn has various ramifications on cardiovascular health and exercise capacity.

## 6. Metabolic Syndrome and Obesity

Obesity is a core element in the diagnostic criteria of MetS, abdominal obesity more so than body mass index. With the increase in obesity, there has been an evident increase in the prevalence of MetS [105]. It is well established that obesity is linked to the many other risk factors that make up MetS, including hypertension, insulin resistance, and hyperlipidemia [27]. Visceral obesity is important to consider when discussing obesity as it has been shown that it correlates with greater muscle sympathetic nerve activity [106,107]. This suggests that visceral or central adiposity may be mechanisms by which sympathetic activity is enhanced and may contribute to the development of additional components of MetS such as insulin resistance. Bergman et al. [108] evaluated whether free fatty acids in visceral adipose tissue are a culprit in the development of insulin resistance and MetS, and whether the anatomical localization of the visceral fat has any significance when it comes to the pathogenesis of MetS. Using a canine model, they concluded that fat-feeding-induced visceral adiposity did lead to primary insulin resistance of the liver [108]. The observed increase in measured free fatty acids, presumably coming from visceral fat, could partially explain the insulin resistance observed in non-diabetic individuals. In humans, Jensen et al. [109] showed that in lean, obese, non-diabetic, and diabetic humans, non-visceral fat in the upper body contributed to most measured free fatty acids. In other words, the excess availability of free fatty acids in the systemic circulation was not mainly from visceral fat.

Whether energy homeostasis complications occur from abdominal or visceral obesity primarily has yet to be determined; however, in either instance, obesity is one of the primary symptoms of MetS and likely one of the first stages of the disease. Being that obesity is typically a condition related to lifestyle and eating habits it provides a low-hanging fruit so to speak for treatment options for the prevention of MetS.

## 7. Autonomic Function: Obesity

Although obesity may be the easiest symptom of MetS to deal with and its subsequent treatment may be a major mechanism in preventing the development of further MetS symptoms it is not without consequences regarding changes in autonomic function [23,49]. For instance, obesity-related metabolic derangements do seem to influence pressor responses [49]. In a study involving normotensive obese females, Prud’homme et al. [49] reported a significant relationship between systolic blood pressure measured during submaximal exercise and various cardiovascular disease risk factors, such as cholesterol levels and waist–hip–ratio. This exaggerated blood pressure response could partially be explained by increased circulating catecholamines in obese patients [34]. The increased sympathetic activity in obesity could also explain the observed elevation in blood pressure [110,111]. In addition to being combined with other factors, obesity has also been shown to independently modify autonomic reflexes. Most notably during exercise, obesity influences autonomic function via increased sympathetic outflow [9] and enhanced vascular resistance [20,22] during muscle metaboreflex activation, in addition to changes to baroreflex sensitivity [5,33]. Furthermore, these changes are not solely related to each individual autonomic reflex, as it is known that muscle metaboreflex pressor responses are buffered by the arterial baroreflex [72,74]. Studies by Dipla et al. [21] and Latchman et al. [112] also showed alterations in both baroreflex function and metaboreflex function in young obese boys relative to their healthy counterparts, suggesting that the effect of obesity is not likely tied to only one autonomic blood pressure control mechanism. Therefore, aside from the physical challenges of exercising with obesity, this evidence suggests that the pathophysiology of obesity contributes significantly to autonomic dysfunction, particularly during exercise. Therapeutic interventions to blunt this pressor response in obese patients could prove beneficial when considering treatments. This was shown by Derella et al. [113], where pressor response to acute stress was blunted with dual endothelin A/B receptor antagonism.

## 8. Effect of Metabolic Syndrome on Autonomic Dysfunction

Sympathetic activity is likely significantly involved in the risk factors of MetS. However, this involvement could have many implications for the integrity of the autonomic nervous system (ANS) and autonomic reflexes. A longitudinal study performed by Licht et al. [114] showed that elevated sympathetic activity does indeed predict metabolic derangements, as the number of MetS components increased over that time period. This indicates that ANS dysregulation could be a predisposing factor to the development of MetS [8,114,115,116]. However, there is also evidence that alterations to the ANS do occur in the setting of MetS and this could potentially be a result of the interplay within the severity of MetS components and/or the addition of components [117].

The sympathetic and parasympathetic nervous systems play an important role in metabolic hemostasis. The sympathetic ANS stimulates hepatic gluconeogenesis while the parasympathetic ANS antagonizes the effects of the sympathetic ANS by promoting glucose storage in the liver and inhibiting hepatic gluconeogenesis [55,118]. Thus, alterations in the total output of either branch of autonomic control likely drive the development of pathologies towards the full diagnosis of MetS through changes in energy homeostasis and energy storage. These changes likely initially occur as a result of lifestyle and dietary choices leading to greater levels of circulating glucose and lipids, which initiate inflammatory pathways that perturb sympathetic homeostasis via changes in blood pressure, insulin, or other factors, and therefore alter sympathetic mediated reflexes.

This ANS imbalance impacts not only metabolic hemostasis and hypertension but also the arterial baroreceptor reflex, leading to orthostatic intolerance and exercise intolerance. Elderly patients with MetS and insulin resistance were found to have reduced baroreceptor sensitivity, suggesting a cardiovascular autonomic imbalance [119]. As Zanoli et al. [120] pointed out, clarifying the role of the neural baroreflex pathway is important in subjects with MetS likely because blood pressure, vascular pathologies, or neuropathy seen in diabetes can have different effects on baroreflex function. Keeping in mind that these pathologies and risk factors are core in the diagnosis of MetS. Furthermore, Zanoli et al. [120] found that baroreflex function is reduced in patients with MetS and this coincided with higher blood pressures, especially as more components of MetS are present.

These changes in baroreflex function inform us that autonomic reflex dysregulation takes place alongside metabolic derangements. It is also clear that the interactions are complex and could be bidirectional. However, it is difficult to elucidate the order of these events. For example, does MetS arise because some individuals are predisposed to autonomic dysregulation and increased sympathetic activity? This may not be completely the case because it does not fully explain obesity, elevated blood sugars, high triglyceride levels, etc., all of which are evaluated when making the diagnosis of MetS. Adding exercise to the equation is necessary for the discussion as it is one of the key components in the clinical battle of MetS, along with a healthy diet and lifestyle. As mentioned, there is already evidence of baroreflex alterations in patients with MetS. In addition, a large study by Sharman et al. [54] noted the association between exaggerated exercise responses in patients with impaired carotid baroreflex sensitivity. While the population in the study did not have MetS, the interaction between the baroreceptor reflex, blood pressure, and exercise reminds us that the individual components of MetS ought not to be studied in isolation.

With alterations to autonomic reflexes in MetS, it is unsurprising that exercise capacity may be compromised. Aside from the physical limitations of exercise in obesity, augmented reflex-mediated increases in sympathetic activity can also limit exercise tolerance. With muscle metaboreflex activation sympathetic tone is raised to the periphery which includes the active muscle from which the reflex originated [62,121]. This creates a positive feedback amplification of sympathetic activity which is even greater in pathophysiological states when baroreflex function is depressed, such as in heart failure [122,123]. Reflex vasoconstriction within the active skeletal muscle would lower oxygen delivery, causing further metaboreflex afferent activation which can increase the sensation of fatigue [124,125,126,127,128]. These findings could explain exercise limitations in patients with obesity and MetS, although further investigation is required to account for the role of other risk factors that comprise the syndrome. For example, muscle metaboreflex activation in patients with type 2 diabetes was shown to result in an exaggerated vasoconstriction [95]. Thus, the pressor response in these patients relied less on cardiac output and more on vasoconstriction and this could also include enhanced vasoconstriction of the active skeletal muscle. While these studies are important in furthering our understanding of the subject, they study only fragments of MetS. It is critical to examine how MetS affects autonomic reflexes, both with and without exercise.

## 9. Metabolic Syndrome Known vs. Unknown Syndrome Component Interactions and Autonomic Function

The two primary models evaluating autonomic function in MetS are human and rat models wherein the former is comprised of multiple variations of MetS components and the latter typically is comprised of four or five of the components of MetS that typically include hypertension. One of the major issues regarding both primary models is the lack of study of the development of components of MetS as each component itself likely plays a role in enhancing or altering autonomic function.

In humans, MetS is a diverse array of components that compromise the syndrome as a whole, and within each collection of components, there are autonomic alterations that arise that are not always uniform. For instance, prior to the development of frank MetS, an autonomic function may be perturbed by obesity, and then the addition of hypertension enhances autonomic dysfunction [4,55,110,129]. Conversely, although hypertension and obesity have both been implicated in autonomic imbalance, studies assessing all of the components of MetS show that they correlate with reduced heart rate variability (HRV) [130], and further, that the highest interaction was between alterations in HRV and plasma glucose levels, suggesting that glucose control has the largest effector of HRV, or that a secondary factor mediates this relationship by exerting significant influences on both factors [131]. Like the previous HRV studies, most multi-component assessments of MetS populations have found that individuals with more existing components of MetS have greater impairment in autonomic function [50,132,133,134,135].

The studies cited above, including the HRV studies, have not classified the phenotypes of MetS, rather they classified subjects based on the number of existing components of MetS or evaluated subjects based on singular components with a given autonomic variable. Few models have taken the approach of examining the changes in different phenotypic expressions of MetS, or how within one subject a combination of components influences overall autonomic function. One recent study sought to evaluate individual component’s contributions to autonomic dysfunction as well as evaluate the phenotype in MetS during exercise and found that the autonomic alterations during exercise could not be explained primarily by any of the three components observed in their phenotypic population [117], thus suggesting it is the amalgamation of components in total as well as potentially other factors that contribute to altered autonomic activity in MetS. Thus, even within studies looking at the severity of the syndrome itself relative to changes in autonomic function across conditions, there are limitations in identifying which aspect of a given MetS phenotype contributes the most to autonomic imbalance.

Overall, the current greatest limitation to determining the primary effector or effectors of changes in parasympathetic and sympathetic activity at rest and during exercise in MetS is the lack of the ability to assess component interactions within the syndrome itself, as well as a current lack of models that evaluate the development of MetS. However, this is not to say that these associations are impossible to determine. Study design regarding MetS observations can be adjusted to enable the assessment of components as they arise or to be able to compare different phenotypic expressions of MetS components and relate changes in autonomic function across these different phenotypic groups.

## 10. Conclusions and Future Directions

Most studies using animal models of MetS did so by isolating select MetS components. However, the complexity of the syndrome and the involvement of many factors suggests a possible common underlying pathogenesis that can better explain why the presentation and etiology of MetS varies in humans. Furthermore, individual factors may interact in complex fashions which may mask individual effects especially when these pathologies develop with different time courses [136]. The diagnostic criteria of MetS are multifactorial, yet not every patient with MetS has the same combination which further complicates understanding of the consequence of MetS. The variation, even among individual risk factors, makes it difficult to elucidate the pathophysiology of this syndrome, as we lack an in-depth understanding of how these individual risk factors interact with each other as well as together as a whole. Further studies designed to elucidate these interactions are likely critical to determining mechanistic treatments for the syndrome.

Exercise is a significant and under-evaluated part of the equation. Exercise is known to have numerous health benefits on cardiovascular health and metabolic well-being. However, exercise itself can lead to exaggerated sympathetic activation when performed in various disease states. Thus, exercise prescriptions should be tailored to maximize the benefits while minimizing the risks. The diagnostic criteria of MetS include risk factors that are measurable and are present and, in many cases, can be prevented. Further studies in patients at risk for, already developing, or already diagnosed with MetS at rest and during exercise are crucial to the development of therapeutic interventions.

## Figures and Tables

**Table 1:** Diagnostic Criteria of Metabolic Syndrome.

Component	Range
Waist Circumference	>102 cm for males or >88 cm for females (>80 cm for Asian females)
HDL Cholesterol	<40 mg/dL for males and <50 mg/dL for females
Blood Pressure	>130 mmHg systolic or >85 mmHg diastolic
Fasted Blood Glucose	≥100 mg/dL
Triglycerides	≥150 mg/dL
